# A Critical Analysis of the CFD-DEM Simulation of Pharmaceutical Aerosols Deposition in Upper Intra-Thoracic Airways: Considerations on Aerosol Transport and Deposition

**DOI:** 10.3390/pharmaceutics16091119

**Published:** 2024-08-24

**Authors:** Georgi H. Spasov, Riccardo Rossi, Andrea Vanossi, Ciro Cottini, Andrea Benassi

**Affiliations:** 1 International School for Advanced Studies (SISSA), 34136 Trieste, Italy; 2 Consiglio Nazionale delle Ricerche-Istituto Officina dei Materiali (CNR-IOM), 34149 Trieste, Italy; 3 RED Fluid Dynamics, 09127 Cagliari, Italy; 4 Chiesi Farmaceutici S.p.A., Largo Belloli, 11A, 43122 Parma, Italy

**Keywords:** orally inhaled drug products, pharmaceutical aerosol, aerosol deposition, CFD-DEM simulation, dry powder for inhalation, human lungs, human respiratory system

## Abstract

The reliability and accuracy of numerical models and computer simulations to study aerosol deposition in the human respiratory system is investigated for a patient-specific tracheobronchial tree geometry. A computational fluid dynamics (CFD) model coupled with discrete elements methods (DEM) is used to predict the transport and deposition of the aerosol. The results are compared to experimental and numerical data available in the literature to study and quantify the impact of the modeling parameters and numerical assumptions. Even if the total deposition compares very well with the reference data, it is clear from the present work how local deposition results can depend significantly upon spatial discretization and boundary conditions adopted to represent the respiratory act. The modeling of turbulent fluctuations in the airflow is also found to impact the local deposition and, to a minor extent, the flow characteristics at the inlet of the computational domain. Using the CFD-DEM model, it was also possible to calculate the airflow and particles splitting at bifurcations, which were found to depart from the assumption of being equally distributed among branches adopted by some of the simplified deposition models. The results thus suggest the need for further studies towards improving the quantitative prediction of aerosol transport and deposition in the human airways.

## 1. Introduction

In recent times, there has been a substantial increase of interest in computational fluid dynamics (CFD) simulations pertaining to the deposition of aerosols in the human lungs, a fact reflected in the growing body of related literature [1,2,3,4]. The applications of these simulations are diverse, encompassing concerns regarding safety, environment, and healthcare. Specifically, these simulations are exploited in diverse areas such as protection from harmful airborne substances, analysis of pathogen spread, and product development in industries like tobacco and pharmaceuticals, i.e., the design of therapeutic aerosols and inhalable drugs [3,5,6,7,8,9]. Such a wide range of applications makes it challenging to establish a unified framework for CFD simulations, as the conditions and parameters can vary significantly from case to case. For example, in the realm of human airways, some simulations consider a tidal breathing condition with minimal turbulence, while others involve a deep breath from an inhaler, resulting in fully turbulent dynamics. Additionally, the size of aerosol particles or droplets varies from nano- to micron-sized, and the temperature of the inhaled air can be either room temperature or higher, depending on the specific application.

As already pointed out in our previous publication [10], even in the simulation of aerosol deposition for specific lung geometries, the reproducibility and comparison of results obtained from different software are, if not impossible, a challenging task due to limited descriptions of the main technical aspects (mesh generations, boundary conditions, numerical integration schemes, etc.), which significantly impact the modeling outcome. Moreover, there exists a recognized absence of comprehensive experimental datasets on airflow and aerosol deposition properties in both extra- and intra-thoracic airways of the same individual, hindering the validation of models to a satisfactory extent.

To ascertain how reliable simulations could be in envisaging the aerosol dynamics and deposition, as required by specific guidelines from pharmaceutical regulatory authorities (e.g., US FDA) [11,12,13,14], in this work, we are specifically addressing the sensitivity of particle dynamics and deposition to the CFD technical aspects mentioned above via a targeted comprehensive simulation campaign. It is worth reminding that our critical analysis was started by first considering the extra-thoracic airways (mouth, throat, and trachea) [15] and was then extended to the upper intra-thoracic ones [10], with particular focus on some of the key aspects in the modeling of the airflow, such as velocity fluctuations, the onset of secondary flows, and the effects of inhaler-dependent inflow characteristics. Here, we adopt a CFD-Discrete Elements Methods (DEM) method to extend the analysis of the impact of these aspects on the global and local depositions in the same patient-specific tracheobronchial geometry.

The paper is organized as follows. Section 2 details all the numerical aspects and the assumptions adopted for the description of both the air and aerosol phases, as well as for their interaction. In the last part of the section, the different possible ways of quantifying aerosol deposition, as used in the literature and in this work, are illustrated and discussed. Section 3 starts with a deep analysis of the impact of the mesh resolution on the total and local particle deposition, and the cause and magnitude of their variability is analyzed. The impact of certain model assumptions on particle deposition is assessed in the subsequent subsections—more specifically: the effect of explicitly modeling or simulating velocity fluctuations, the impact of different boundary conditions to drive the airflow, and the use of different types of air plumes entering the patient mouth and driving the injected particles. The section concludes with a comparison of our results with the data presented by other authors in both experimental and simulation works. Lastly, in Section 4, we draw our conclusions and illustrate, in our opinion, the next steps necessary to achieve quantitative and reliable aerosol deposition predictions.

## 2. Materials and Methods

Before focusing on the numerical aspects of the aerosol dynamics, this section summarizes the details about the adopted lung geometry and the simulation of the airflow. The former has already been employed and the latter deeply discussed in our previous studies [10,15]. The section concludes with a description of the different methods to quantify aerosol deposition adopted throughout the paper.

### 2.1. Adopted Lung Geometry

**Figure 1:**
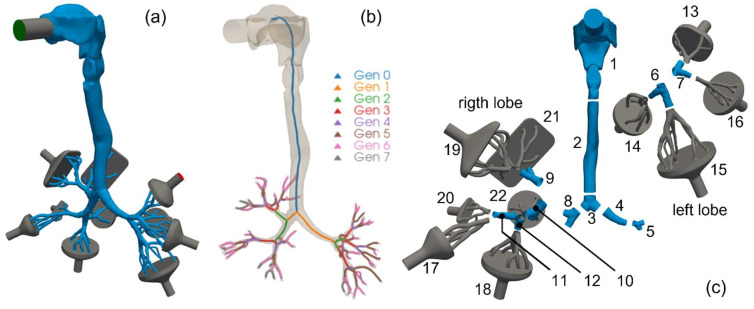
Lung geometry adopted in this study. (**a**) Sketch of the mouth–throat tract and upper airways, including the inlet inhaler pipe and funnel-shaped plenums at the outlets. (**b**) Same geometry with each airway colored according to its generation number. (**c**) Sketch of the geometry segmentation adopted for the quantification of local aerosol deposition.

The present study exploits the SimInhale reference geometry, for which a significant amount of experimental characterization and computational modeling have been made publicly available [16,17,18,19,20]. The geometry, shown in Figure 1a, is obtained combining data from different adult healthy males and includes airways up to the 7th generation [21]. The green patch represents the air and particles inlet and is connected to the mouth through a cylindrical pipe representing the terminal part of an ideal inhaler. The bifurcations pertaining to different lobes have been grouped to enter the same funnel-shaped plenum, the ten red patches being the system outlets. The gray-colored surfaces are plastic- or metal-made, and thus, the simulated aerosol particles bounce away when colliding with them, whereas the blue-colored surfaces are wet or covered with mucus and the aerosol particles stick to them upon collision and are considered deposited. In a closer inspection, generations 6 and 7 are not entirely mapped, as visible in Figure 1b, where the branching airways are colored according to their generation number. Finally, Figure 1c shows the partition of the whole tracheobronchial tree in 22 different segments, where the simulated local aerosol deposition is quantified in accordance with the original deposition experiments by Lizal et al. [17].

### 2.2. Airflow Modeling

The airflow dynamics is described through a Reynolds-averaged Navier–Stokes (RANS) formulation of the governing equations and solved on a hexahedral-dominant mesh using second-order accurate spatial discretization schemes with the open-source code *OpenFOAM* [22,23], based on the unstructured finite volume method. Turbulence is modeled via the k−ω SST approach [24] for both steady-state and time-dependent simulations. Low Reynolds wall functions are employed to explicitly resolve the near-wall region of the turbulent boundary layer up to the viscous sublayer.

The generation of the computational mesh of the intra-thoracic airways can be particularly challenging due to irregularities in their geometry and their different orientation and length scale. In our previous study, focused on the airflow dynamics, we generated five different meshes with increasing sizes of 3.6, 5.3, 8.1, 11.7, and 14.6 million elements and different refinement criteria [10]. In the first part of this work, we adopt the same set of meshes to evaluate the impact of mesh size and refinement on aerosol deposition. Subsequently, while focusing on the influence that certain approximations/assumptions on the airflow play on particle deposition, we will consider a fixed mesh size of 8.1 M elements.

The airflow is driven by imposing proper boundary conditions onto the inlet and outlets of the simulated tracheobronchial tree portion. In our previous study, we verified how, imposing a steady 60 L/min flow at the mouth (inlet-driven simulations), the flow splitting and ventilation of the tree are different compared to the experimental data [10]. Conversely, driving the system uniquely from the outlets, i.e., imposing a priori the experimentally measured flow splitting in the different lobes (outlet-driven simulations), grant the correct flow splitting, but the turbulent kinetic energy density profile k, developing at the inhaler outlet, is inconsistent with the experiments and high-fidelity simulations. We demonstrated that the only way to reproduce the correct airflow behavior, consistent with the experimental data, is to simultaneously impose the airflow velocity profile at the mouth while fixing the flow splitting in 9 of the 10 outlets (mixed-driven simulations). Therefore, in the present study, we adopt the latter approach, except when we explicitly compare the results with inlet-driven simulations to show how large and relevant the impact of a different ventilation on the quantification of local aerosol deposition can be. Lastly, in our previous analysis, we evaluated how deep in the lungs the airflow retains memory of the original inhaler-dependent structure it had while entering the mouth. To this aim, we performed simulations with a one-directional, turbulent pipe-like flow, as well as with a swirled flow. In the present work, most of the simulations are performed adopting a one-directional, plug flow in line with the reference experimental data. Only while exploring the effects of different inhaler types on deposition in deeper airways will we compare the one-directional and swirled flow results. All the numerical and implementation details of the different driving boundary conditions, as well as the different types of inflow at the mouth, are described in Spasov et al. [10].

In the time-dependent simulation, the integration step $\Delta t_{f}$ has been selected to keep the fluid Courant number $Co_{f}$ between 1 and 5. For the 8.1 M elements mesh, this leads to $\Delta t_{f}=5\times 10^{-5}\;s$. A deep discussion on the adequacy of this value to sample the explicit time oscillation of the simulated flow was previously considered [10,15].

### 2.3. Aerosol Modeling

The solid aerosol phase is simulated through the native Lagrangian particle tracer of *OpenFOAM*. The particles are assumed to be spherical, with a diameter d=4.3 μm, as in the reference experiment, or vary in the range 1–10 μm to investigate the effect of particles size on aerosol deposition. The particle material density has been set to $\rho_{p}=914\;kg/m^{3}$, a value typical of the diethylhexyl sebacate particles employed in the deposition experiments [17]. Particles equations of motion are integrated in time using a first-order implicit Euler numerical scheme. The coupling with the fluid is unresolved, i.e., the aerosol is represented as an ensemble of point particles, and more than one particle can populate the same CFD mesh element. The particle–air interaction occurs through the single-sphere drag force in the Putnam formulation [25], pressure gradient, and gravitational forces. The coupling is assumed to be one-way only, i.e., the loss of momentum experienced by the fluid in dragging the particles is negligible; thus, the presence of the solid transported phase does not influence the fluid one. Such an assumption is certainly valid for diluted aerosols entering the patient mouth, e.g., environmental aerosols, or drug product administration through diluted nebulizers, but it must be dropped in the case of large-dose dry powder inhalers (see, for instance, Appendix A in Spasov et al. [15] for an estimation of the aerosol density in the mouth for different realistic drug product doses). The one-way coupling is also not adequate when the full inhaler is incorporated in the computational domain to simulate explicitly the drug dose aerosolization. In fact, in dry powder inhalers (DPIs) the drug dose is initially at rest before aerosolization, collected in a cup or a capsule, where it reaches a very high local density. In carrier-based DPIs, large particles accumulate in the edges of the inhaler, significantly rising the density there (for a study of the effect of one-way and four-way couplings in DPIs, see, for instance, Ponzini et al. [26]). In pressurized metered dose inhalers and soft mist inhalers, aerosol droplets are generated in a cone-shaped spray; in the cone tip, the density of the liquid phase can reach very large values, thus requiring a four-way coupling scheme [27]. Again, in the spirit of highly diluted aerosol, particle–particle collisions are neglected. Upon colliding with a boundary wall, particles can bounce back elastically (in the inhaler pipe and funnel-shaped plenums) or stick and remain in place (in the wet airway walls).

The value of the air velocity to be used in the drag force calculation must be derived from the vectorial field computed on the CFD mesh, depending on the instantaneous particle position. *OpenFOAM* allows for two different strategies to interpolate the Eulerian velocity field to the position of Lagrangian particles: the *cell* option uses the air velocity value corresponding to the mesh element occupied by the particle at any given time; this can cause discontinuities as the particle moves from one mesh element to the nearby one. Using the *cellPoint* option, the air velocity value is interpolated between the neighboring mesh elements based on the particle position. The latter option is adopted for all the simulations presented in this paper. A calculation with the *cell* option is presented, for the sake of comparison, in the section where we assess the impact of velocity interpolation on local particle deposition.

The aerosol particles can be injected into the computational domain using different strategies through the inlet patch to then track their trajectory up to the impact with the sticking walls or upon reaching the outlets. In the first strategy, named the *patchInjection* model, widely employed in this work, particles are inserted in the face center of each mesh element belonging to the inlet patch. This choice makes the particle insertion random but mesh-dependent, since the particle’s injection position will change with the mesh resolution and refinement strategy. Conversely, the *manualInjection* model, used in this work for the sake of comparison with the *patchInjection* model, requires the user to manually define the insertion locations and the mass fraction of particles to be inserted through each of those locations. When using the latter option, we defined a regular lattice of insertion points to have a uniform injection over the inlet patch now independent on the mesh resolution.

For the steady-state simulations, the integration time step has been set to $∆t_{p}=10^{-6}\;s$, a value one order of magnitude smaller than the smallest particle relaxation time $\tau ≅6\times 10^{-5}\;s$ estimated for particles with d=4.3 μm. In the time-dependent simulations, the particle integration step is automatically set by the software based on the formula $∆t_{p}=Co_{p}\cdot \Delta t_{f},$ where $Co_{p}$ is the particle Courant number. Setting $Co_{p}=5\times 10^{-3}$ and having $\Delta t_{f}={5\times 10}^{-5}\;s$, we obtain $\Delta t_{p}={2.5\times 10}^{-7}\;s$, a value again one order of magnitude smaller than the smallest particle relaxation time (used for the time-dependent simulation particles in the range 1–10 μm; the relaxation time range is $\tau \;\sim \;3\times 10^{-6}–3\times 10^{-4}\;s$).

As the RANS simulations neglect the velocity fluctuations, and the time-dependent RANS simulations only capture them partially, the particle–eddy interaction must be included in the model through an effective stochastic term. In our simulations, we employ the general purpose Discrete Random Walk (DRW) already implemented in *OpenFOAM* [28]. The adequacy of such a simple model has been debated by several authors, and some of them believe it leads to an overestimation of small particle deposition [29,30,31]; others attribute the overestimation to a too-coarse sampling of the turbulent kinetic energy density k field near walls [5]. In our recent analysis [15], we obtained results in line with the second hypothesis and demonstrated that a fine enough sampling of k close to the bounding wall does not lead to deposition overestimation.

### 2.4. Aerosol Deposition Metrics

Several ways have been defined and adopted in the literature to quantify aerosol deposition in both experiments and simulations. In this work, we will make use of three of them:

- **Total Deposition Fraction:** the ratio of the amount of aerosol particles/mass sticking onto the inner walls of the cast/computational domain to the total amount of particles/mass of the aerosol that have been injected into the cast/computational domain $N_{tot}$:
  $$TDF=\sum_{i=1}^{22}N_{i}/N_{tot}$$
  with $N_{i}$ the number of particles sticking in the ith section of the cast. This quantity conveys information on the total stopping power of the whole geometry with no local information.
- **Deposition Fraction:** the ratio of the amount of aerosol particles/mass sticking onto the inner walls of each individual segment of the cast/computational domain $N_{i}$ to the total amount of particles/mass of the aerosol that have been injected into the cast/computational domain $N_{tot}$:
  $${DF}_{i}=N_{i}/N_{tot}$$
  It conveys information on how much of the total injected aerosol/drug dose has been stopped by each portion of the tracheobronchial tree.
- **Deposition Efficiency:** the ratio of the amount of aerosol particles/mass sticking onto the inner walls of each individual segment of the cast/computational domain $N_{i}$ to the amount of particles/mass of the aerosol that entered such individual segment of the cast/computational domain $N_{i}^{in}$:
  $${DE}_{i}=N_{i}/N_{i}^{in}$$
  It provides a measure of the stopping powder of each individual portion of the tracheobronchial tree.

## 3. Results

As a first step in our analysis, in Section 3.1, we provide evidence of how strongly the calculated aerosol deposition depends on the CFD mesh resolution. Several tests are presented to exclude other possible sources of uncertainty and numerical errors. In Section 3.2, we quantify the influence of time fluctuations of the air velocity on aerosol deposition by comparing time-dependent calculations with steady-state simulations. The impact of different flow driving conditions, i.e., of different flow splitting and ventilation anisotropy, on the deposition is dealt with in Section 3.3. In Section 3.4, we modify the structure of the inflow at the mouth, mimicking different inhaler types, to evaluate how deep in the lower airways deposition can be influenced, i.e., how deep in the lungs the aerosol retains memory of its initial condition while entering the patient mouth. The last section concludes the analysis by comparing our deposition results with those measured or calculated by other authors.

### 3.1. Mesh Sensitivity of Calculated Aerosol Deposition

**Figure 2:**
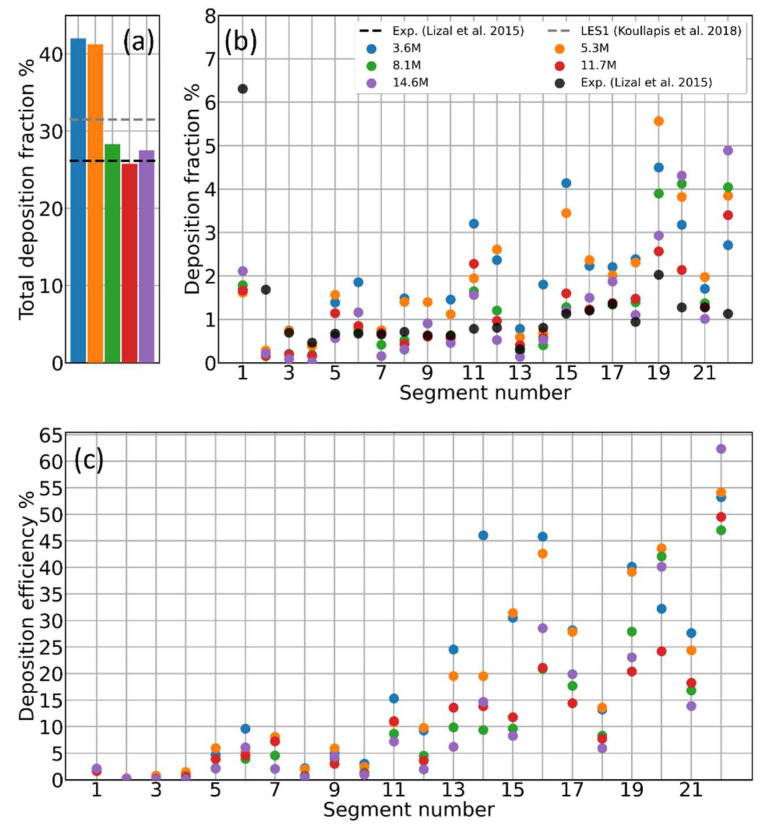
Aerosol deposition as a function of the CFD mesh resolution. (**a**) Percent total deposition; black and gray dashed lines represent, respectively, the experimental reference value from Lizal et al. [17] and the simulated value by Koullapis et al. [16]. (**b**) Percent deposition fraction as a function of the segment index; black dots represent the experimental reference value from Lizal et al. [17]. (**c**) Percent deposition efficiency as a function of the segment index. Segment indexing is shown in Figure 1c.

Mesh sensitivity analyses are usually performed to ensure the physical quantities calculated through CFD simulations and do not depend significantly on the chosen spatial discretization, a necessary yet non-physical numerical artifact [7,32,33,34,35,36,37,38]. Many computed quantities usually converge to mesh-independent values as the mesh resolution increases, i.e., the spatial discretization improves. When this is the case, one can adopt a certain mesh resolution based on the best compromise between uncertainty on the physical quantity of interest and computational cost. We indeed found this kind of situation when performing a mesh sensitivity study on the total deposition fraction for d=4.3 μm aerosol particles, injecting 100 K particles. This first simulation campaign has been performed on a steady-state airflow, neglecting, for the moment, the time fluctuations of the airflow and particle–eddy interaction. Figure 2a shows the total deposition fraction for five increasing mesh sizes converging nicely to the experimental value by Lizal et al. [17]; the Large Eddy Simulation (LES) result by Koullapis et al. [16] is also reported for comparison. The histogram may suggest a satisfactory agreement when the experimental value is already reached with an 8.1 M elements mesh. However, when looking at the local deposition fraction for each segment of the cast, the previous picture changes, as displayed by the histograms in Figure 2b. Given that these preliminary simulations neglect a few important physical ingredients, one should not expect the numerical deposition values to converge to the experimental ones (black dots). However, one should at least notice, in principle, a progressive reduction of the discrepancy between the histograms by increasing the mesh resolution. This is not the case: the deposition fraction for the different segments is fluctuating with no converging trend with respect to the mesh resolution. By plotting the deposition efficiency histogram, the situation remains the same; see Figure 2c. For the upper segments (i.e., the extra-thoracic airways, the larynx, and the first bifurcation), all the meshes seem to lead to the same deposition percentage; however, the values for the deeper segments appear to again be scattered, with no apparent correlation with the mesh resolution. Notice how, with both the deposition normalizations, the discrepancies increase more and more as one progresses from the upper to the lower segments. This suggests that the deposition statistics are far from convergence due to an insufficient number of particles reaching the lower generations. Indeed, most of the particles cross the upper generations deciding whether to stick there or not, but due to sticking and the subsequent branching, the deeper ducts see only a small fraction of the total injected particles. To find which is the smallest number of injected particles necessary to consider the deposition histograms converged, we progressively increased the number of injected particles up to 150 k on the 8.1 M elements mesh, as done by other authors [37,39,40].

**Figure 3:**
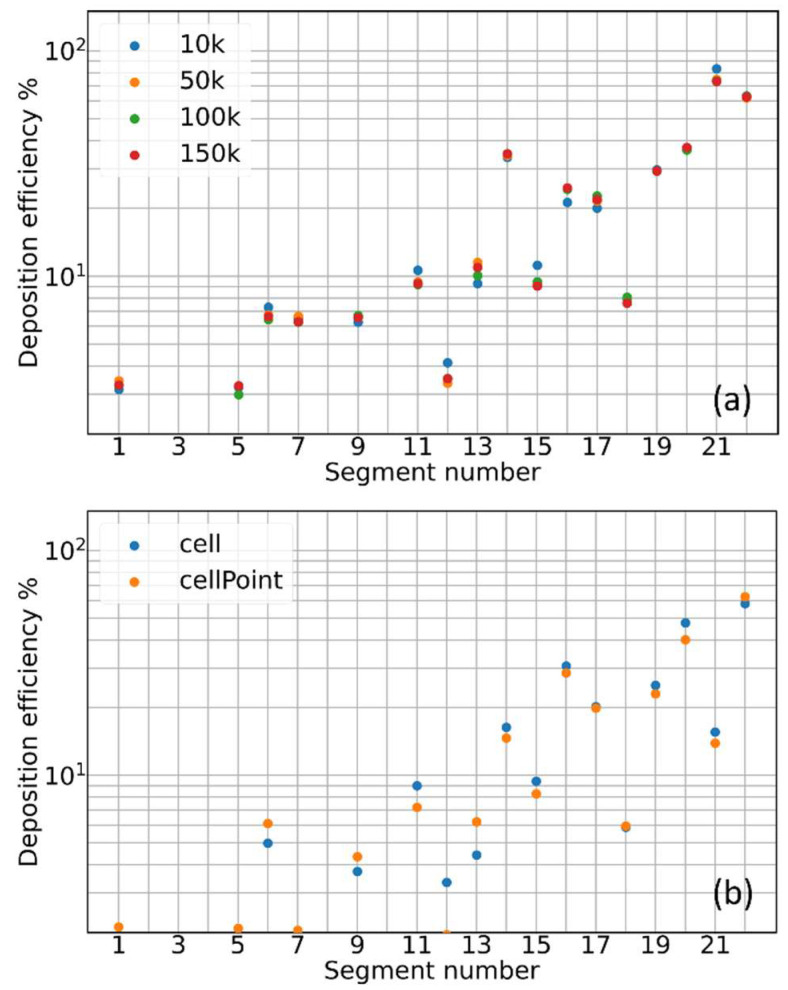
Percent deposition efficiency as a function of the segment index for (**a**) different numbers of injected particles and (**b**) different air velocity interpolation methods. Segment indexing is shown in Figure 1c.

The same is true for the deposition fraction that, by definition, should be even less sensitive to the particle number. Testing different time integration steps and different injection methods, the deposition efficiency histogram for the 8.1 M elements mesh remains substantially unchanged with respect to Figure 2c and Figure 3a. Changing the diameter of the injected aerosol particles up to 10 μm does not change the picture; deposition histograms do not converge or show any ordered trend with increasing mesh size (all these results are documented in the Supplementary Materials). A slightly more significant impact on deposition is due to changes in the air velocity interpolation scheme. Figure 3b reveals how differences of 1–2% in the percent deposition efficiency emerge by switching off the interpolation scheme (*cellPoint*) in favor of a single-cell air velocity value (*cell*). This finding suggests how the origin of the lack of convergence for the deposition histograms might be of a numerical nature, although interpolation alone cannot explain the much larger discrepancies shown in Figure 2.

**Figure 4:**
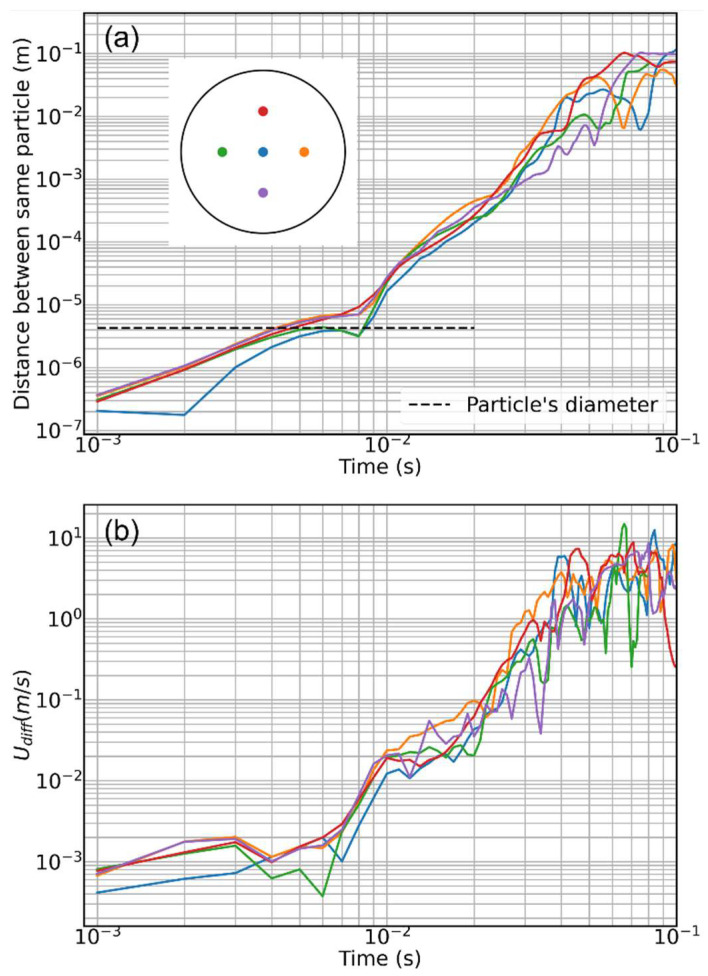
The diverging particle trajectories problem. (**a**) Modulus of the difference between the position vector of the same particles in two simulations, with 8.1 and 14.6 M mesh elements, as a function of time. The different colors represent five particles injected in the different points of the inlet patch, as illustrated in the inset. The black dashed line marks a distance between the two trajectories of the same particle equal to the particle diameter. (**b**) Modulus of the difference between the velocity vector of the same five particles in the same two simulations.

To further investigate the problem, we concentrated on five particles only, color-coded and positioned to form a cross on the inlet patch, as displayed in the inset of Figure 4a. Their trajectories have been recorded for two steady simulations with 8.1 and 14.6 M mesh elements, respectively. For each particle, the difference in position and velocity (modulus) at each time step of the two simulations is plotted in Figure 4a,b. Despite traveling along different regions of the mouth and throat, all the particles behave in the same way: when still in the central part of the mouth, the trajectory discrepancy between the two simulations starts to be comparable to the particle diameter (black dashed horizontal line in Figure 4a; after traveling a few centimeters in the mouth, the two trajectories of each particle are already diverging. The same is true for the particle velocity difference between the two simulations. The divergence process is continuous and fast, driven by the power law behavior. Notice how, in the early instants of the tracking, the difference in the relative positions of the particles is much lower than the discrepancy in the particle velocity. Clearly, for both the simulations analyzed in Figure 4, we verified that the chosen mesh resolution does not significantly alter the fluid dynamics structures and the secondary flows. In fact, it has been recently demonstrated how secondary flow structures/vortexes developing in the mouth and throat volume, especially close to the irregular boundaries, can depend on the adopted mesh resolution if the latter is not large enough [15]. Although completely identical in shape and structure, the air velocity field remains weakly dependent, in intensity, on the specific adopted mesh and its refinement close to the bounding walls [10]. Here, we are referring to variations in the air velocity magnitude in the order of 2%, which, however, seems able to produce significant effects on aerosol transport and in determining the deposition statistics.

One point that deserves further investigation relates to the use of higher-order integration schemes for the aerosol particle dynamics, e.g., the second-order Velocity Verlet scheme. So far, higher-order algorithms have never been implemented in *OpenFOAM*, preventing us from understanding if and how they could mitigate the mesh dependency of aerosol deposition statistics. Using different software, other authors have presented results obtained with higher accuracy numerical integration schemes for the Lagrangian phase [41]. However, those analyses never discuss the impact of different numerical schemes on aerosol particle dynamics and deposition.

**Figure 5:**
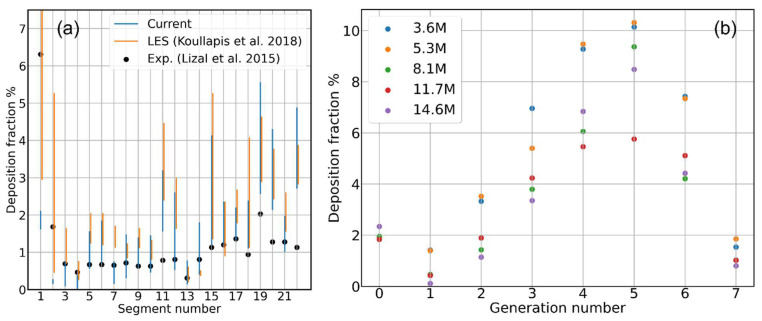
(**a**) Percent deposition fraction as a function of the segment index, and comparison of the present simulation results with the computational work by Koullapis et al. [16] and with the experimental work by Lizal at al. [17] for the SimInhale model. (**b**) Percent deposition fraction as a function of the airway generation number for the five different meshes adopted in this study.

Despite the large variability due to the mesh dependence, a comparison with the SimInhale reference deposition data is still possible. In Figure 5a, the simulated deposition fraction for each segment is represented by a blue vertical bar connecting the highest to the lowest deposition values obtained with the different meshes, i.e., connecting the highest to the lowest colored dots in Figure 2b. A similar plot can be made for the LES and RANS simulations by Koullapis and coworkers [16], as they presented three different simulation results for each computational method, differing for many numerical details, one of which was always the mesh resolution. The LES results variability is plotted with yellow bars in Figure 5a. It is indeed as large as in our case, and both numerical studies tend to overestimate the experimentally observed deposition fraction (black dots) in the lower generations [17]. The same is found comparing the RANS results by the same authors, here omitted to keep the plot readable. One possible interpretation, which deserves further investigation, is that even the Koullapis deposition simulations suffer from the same problem of mesh dependence affecting our own data, with the other differences in numerical schemes playing only a marginal role.

To conclude, increasing the mesh resolution, the total aerosol deposition converges, with 5% of uncertainty, to a value in close agreement with the experiments, while, for local deposition, the dependence on the adopted mesh resolution remains an open issue, with some segments showing uncertainties up to 30%. One might thus be led to the conclusion that spatially integrated quantities converge faster and easier than spatially distributed ones and wonder if an intermediate solution exists where some integration ensures convergence without completely losing the information on aerosol distribution. With this idea in mind, we regrouped the deposition fraction data based on generation rather than geometry segments (details on how each deposited particle is assigned to a specific generation can be found in the Supplementary Materials). This generation-wise representation increases the level of integration compared to the complete distribution over the 22 segments of the full geometry but does not reduce it to a single number, like the total deposition fraction, preserving the information on how different generations are interested by particle deposition. The resulting histogram is shown in Figure 5b; unfortunately, the same lack of convergence with the increasing mesh resolution is clearly visible, as here, the uncertainty remains to the order of 25%, still smaller than the segment-wise one. We also computed more integrated deposition datasets presented in the Supplementary Materials: (i) the sub-lobar deposition fraction, grouping the mouth–throat tract, and the five sub-lobes of the left and right bronchi, with an uncertainty of 20%, and (ii) the lobar deposition fraction, grouping only the mouth–throat tract and the left and right bronchi, with an uncertainty of 13%. It is thus clear that the uncertainty among the higher resolution meshes decreases smoothly only by the effect on spatial integration.

### 3.2. Contribution of Resolved and Modeled Time Fluctuations on Aerosol Deposition

**Figure 6:**
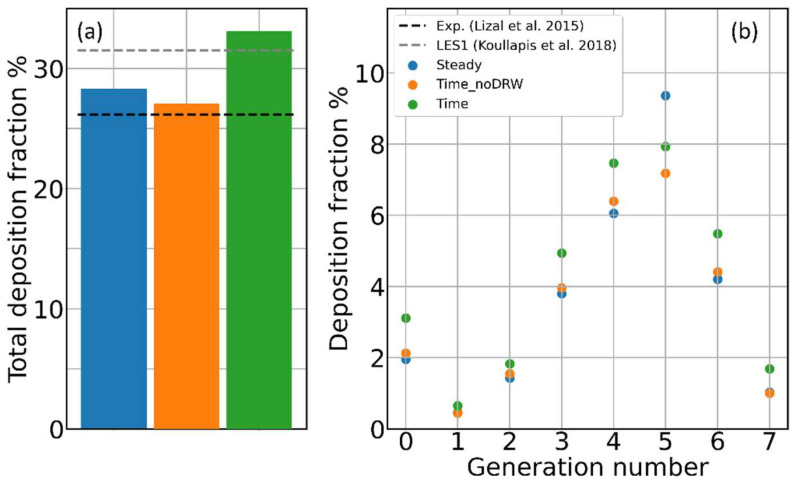
Aerosol deposition and time fluctuations of the airflow. (**a**) Percent of the total deposition for a steady simulation of time-dependent simulations without and with DRW, respectively. Black and gray dashed lines represent, respectively, the experimental reference value from Lizal et al. [17] and the simulated value by Koullapis et al. [16]. (**b**) Percent deposition fraction for the same cases in panel (**a**) as a function of the airway generation number.

So far, we have dealt only with steady-state simulations, neglecting, on purpose, velocity fluctuations in time and the particle–eddy interaction. In this section, limiting ourselves to the 8.1 M elements mesh, we explore the effect of time fluctuations on particle deposition. As we already discussed in previous works [10,15], in our low-Reynolds regime, time-dependent RANS simulations can explicitly capture a significant part of the air velocity fluctuations [42,43,44]. However, Figure 6 reveals how their inclusion affects by only a few percent both the total and generation-wise deposition fractions. As discussed in the Materials and Methods section, DRW-based approaches can be used to replace the particle–eddy interaction for that part of the turbulence fluctuations implicitly accounted for through the k−ω SST model. Including a DRW in our simulations, the total deposition increases by 6–7%, becoming slightly higher with respect to the experimental value but closer to the LES simulations. In conclusion, the particle–eddy interaction seems to be relevant if one is interested in quantitative deposition calculations; however, both the time-dependent RANS and LES approaches are found to slightly overestimate it. Interestingly, in both the approaches, part of the particle–eddy (or particle–turbulence) interaction is captured explicitly, and part is modeled.

### 3.3. Role of Flow-Driving Strategies on Aerosol Transport and Deposition

**Figure 7:**
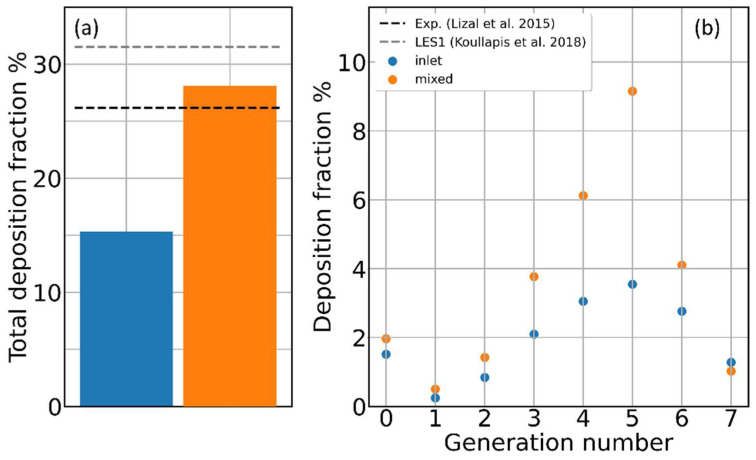
Aerosol deposition and airflow driving conditions. (**a**) Percent of the total deposition for the inlet-driven and mixed-driven airflows; black and gray dashed lines represent, respectively, the experimental reference value from Lizal et al. [17] and the simulated value by Koullapis et al. [16]. (**b**) Percent deposition fraction for the same two simulations of panel (**a**).

**Figure 8:**
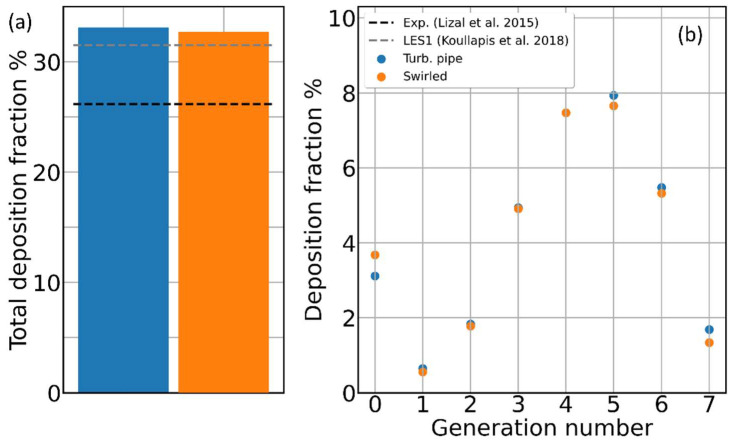
Aerosol deposition and different airflow types. (**a**) Percent of the total deposition for the straight turbulent pipe flow and swirled airflow; black and gray dashed lines represent, respectively, the experimental reference value from Lizal et al. [17] and the simulated value by Koullapis et al. [16]. (**b**) Percent deposition fraction for the same two simulations of panel (**a**).

The importance of selecting proper boundary conditions to drive the airflow has been addressed by a few authors [45,46,47,48], and they have all demonstrated how the airflow splitting at every bifurcation—and thus, the whole ventilation anisotropy—are extremely sensitive to the imposed pressure drops/flow rates at inlet/outlets of the bronchial tree. To the best of our knowledge, however, only Hayati et al. [45] have discussed how strongly this choice reflects on particle transport and deposition. To this aim, we performed steady-state deposition calculations with both the inlet-driven and mixed-driven airflow conditions, previously described in the Materials and Methods section. Again, for this analysis, we concentrated on the 8.1 M elements mesh and on the 4.3 μm particles only. By adopting an inlet-driven condition, the total deposition in the whole geometry is almost halved (see Figure 7a). Comparing the generation-wise deposition fraction for the two driving modes, illustrated in Figure 7b, discrepancies by more than a factor of two are visible in the fourth and fifth generations. The reason for such large discrepancies is different airflow splitting at each bifurcation and the corresponding and correlated different particle splitting. Histograms of the airflow and particle splitting at each bifurcation for the different driving conditions are illustrated in Figure 8. While, for the first three bifurcations, the differences between the inlet-driven and mixed-driven flows are minimal, less than 5%, in deeper segments, the differences can be 10–15% and higher.

Two other important points arise from the observation of Figure 8: (i) the airflow splitting is always far from being 50–50% for the two bifurcations, as, in the upper ones, it is always around 30–70%, gradually reaching 40–60% for the deeper ones, and (ii) although clearly correlated to airflow splitting, particle splitting can quantitatively differ from the latter; in the first bifurcation analyzed in Figure 8, the particle and flow splitting are almost identical; however, for certain bifurcations, differences up to 10% are visible. In their need of simplifying the air and aerosol dynamics, many of the simplest, yet most used, whole lung models assume airflow splitting proportional either to the fraction of the daughter to the mother branch distal volume [49] or to the cross-section of the two daughter branches [50]. The aerosol particle splitting can be assumed to be proportional to the airflow slitting [51] or estimated based on the bifurcation geometry parameters [52]. While, at present, they are not able to verify the hypothesis on airflow splitting, CFD simulations can be used to have better insights on the particle splitting. More generally speaking, CFD and CFD-DEM simulations could be used to estimate the error in the calculated aerosol deposition due to our poor knowledge of the physiological/realistic ventilation anisotropy or due to our difficulty in imposing it when modeling lung deposition at different levels of approximation and/or when the models focus only on a portion of the bronchial tree.

### 3.4. Effect of Inhaler Flow Type on Aerosol Deposition

**Figure 9:**
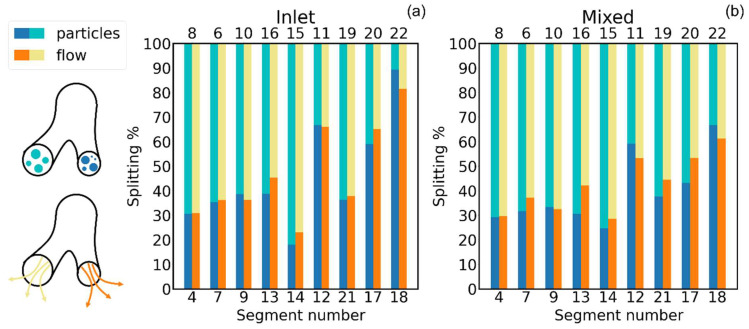
Airflow and aerosol particle splitting at each bifurcation. (**a**) Inlet-driven airflow case and (**b**) mixed-driven conditions case. In both cases, each column of the histogram represents a bifurcation splitting two segments of the model geometry, and the segment numbers are indicated above and below the column. The blue and green colors indicate the percent aerosol particle splitting between the left and right branches of the bifurcation, and the orange and yellow colors indicate the corresponding percent splitting of the airflow crossing the same bifurcation.

Even when having a similar resistance, different types of DPI inhalers can generate very different types of airflow inside the patient’s mouth. For example, the presence of cylindrical geometries and tangential inlets favor the formation of swirling flows at the inhaler outlet, while the insertion of a grid, besides increasing the turbulence, rectifies such swirling flows [32,40,41,53,54,55]. How a different shape of the air plume developing in the patient mouth, at the same airflow rate and aerosol composition, influences the deposition and to which depth in the bronchial tree remain poorly investigated problems. In a previous work focusing on the airflow only [10], we compared time-dependent simulations of a straight turbulent pipe flow and a swirled flow (with swirl number 0.75 taken from a realistic DPI inhaler [26,56]). We found that, while the average air velocity in all the generations is practically identical, the velocity fluctuations are, on average, 11% larger for the swirling flow case and extend deep to the lowest generations. The effect of such a difference in velocity fluctuations on the aerosol deposition due to the different inflow types is illustrated in Figure 9; both the total and generation-wise deposition look very similar. Notice that, applying the same driving conditions, both the airflow and particle splitting at each bifurcation look very similar for both the inflow types. In absence of any significant differences between the inflow types, other than time fluctuations of the air velocity, we can conclude the latter has no impact on fine aerosol deposition in the intra-thoracic airways. Clearly, this is true for integrated quantities such as the total and generation-resolved deposition, but as we have already shown for the extra-thoracic airways [15], the inflow type can significantly affect the local deposition spots and pattern.

### 3.5. Deposition for Different Aerosol Particle Sizes

**Figure 10:**
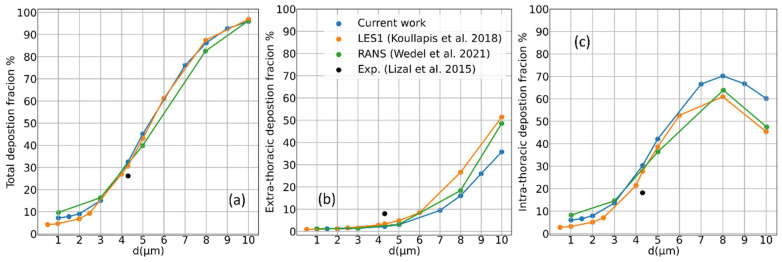
Aerosol deposition as a function of the particle size. (**a**) Total percent deposition fraction in the whole SimInhale geometry. (**b**,**c**) Extra-thoracic and intra-thoracic percent deposition. Our calculations are represented in blue against previously published simulation data, green [5] and yellow [16], and deposition measurements in black dots [17].

In the analysis performed so far, we kept the size of the injected aerosol particles constant at d=4.3 μm, as this is the value adopted in the reference experiments. In this last section, we discuss how the particle deposition changes with the d and compare our results with the calculations presented by other authors. Figure 10a shows the total deposition data for the time-dependent simulations; with the particle diameters in the range of 1–10 μm, the typical S-shaped profile is found, and the agreement with the calculations by Wedel at al. [5] and Koullapis et al. [16] is remarkable. A slight discrepancy is found when comparing the deposition in the extra-thoracic and intra-thoracic airways only; see Figure 10b,c, as our simulations tend to underestimate the intra-thoracic one. The small discrepancy with respect to the deposition experiment is the same for all the simulations.

## 4. Conclusions

In this work, we presented a study of aerosol deposition in the intra-thoracic airways using a coupled CFD-DEM method. The analysis was based upon our previous study of the same patient-specific tracheobronchial geometry [10], focused on airflow considerations, which followed the analysis of airflow and deposition in the extra-thoracic airways [15] of the same patient using the same numerical model. These studies represented, overall, an effort towards evaluating the reliability and accuracy of computer simulations to predict aerosol deposition in the human respiratory system, for which we provide, in this section, the main conclusions and highlight the remaining open issues for practitioners in the field.

The lack of convergence of local aerosol deposition with increasing the mesh resolution remains the most remarkable open issue. We have demonstrated how the uncertainty associated with the mesh resolution reduces more and more as one integrates the local deposition over generations, sub-lobes, lobes, etc., leading to an apparent convergence of the total deposition only. This numerical issue remains mostly neglected in the literature. Many authors have checked the convergence of their simulations against only the airflow properties [7,32,35,36,38,57], while some others have verified the convergence of the total deposition only [39,40,48,58,59], and still others have verified the convergence on both [33,34,37]. From our analysis, it seems the discrepancy of the local deposition among the different meshes is deterministic and well reproducible, persistent for all particle sizes in the explored range of 1–10 μm. It does not change with the number of injected particles, provided at least 50 k particles are used, and it does not depend on the injection position nor on the interpolation scheme adopted to calculate the particle–air interaction. The issue seems related to the particle integration algorithm itself; unfortunately, further analysis is necessary to confirm this conclusion, as *OpenFOAM* features a first-order time integration algorithm only. The same identical particle trajectory evolution, on top of the same fluid dynamics simulation, should be performed using higher order numerical integration schemes, such as the second-order Velocity Verlet or the fourth order Runge–Kutta. If the discrepancy in particle deposition will attenuate when significantly increasing the order of the integration scheme, then our hypothesis will be confirmed.

Another simulation parameter found to have a major impact on particle splitting and deposition in the bronchial tree is the ventilation anisotropy, induced and controllable through the applied boundary conditions. Even a simple in vitro deposition experiment with a constant flow rate can be difficult to simulate correctly if the flow rate at the different outlets has not been measured experimentally and cannot be imposed through the boundary conditions. Therefore, even in the presence of realistic inhalation profiles and subject-specific bronchial tree geometries, the predicted deposition can disagree with the measured one, if incorrect flow-driving conditions are adopted. Quantitative deposition simulations in physiologically realistic conditions can only be achieved if the subject-specific ventilation anisotropy can be measured and imposed in the simulations. In this respect, functional imaging is promising [60,61].

A less impacting but still relevant role, aiming at quantitative deposition calculations, is played by the fluctuations of the air velocity. Time-dependent RANS simulations can capture part of these velocity fluctuations, explicitly altering the local deposition calculations compared to the steady-state counterpart. A similar effect is obtained by adding the stochastic force representing the missing part of the particle–eddy interaction due to the presence of a turbulence model. With the adopted typical flow rate of 60 L/min, the magnitude of the modifications in the local deposition are comparable and of the order of 6%. Ideally, the RANS approach should be dropped in favor of higher fidelity methods such as Large Eddy Simulation or Direct Numerical Simulation (DNS) able to better capture the temporal and spatial correlation of the turbulent eddies, as well as their interaction with aerosol particles. This is, however, possible only when the flow rate and, thus, the Reynolds number of the flow are small enough to keep the spatial resolution of the mesh computationally affordable. Around and below 30 L/min, i.e., in typical tidal breathing conditions, a LES approach is certainly feasible. When simulating single strong inspiratory acts from an inhaler, 60–80 L/min flows develop, and the mesh resolution should be increased significantly, considerably enhancing the computational cost and the simulation duration; this fact severely limits their exploitation in applied/design contexts. Moreover, the particle time integration step must carefully be selected based on the smallest particle–fluid interaction scale [62]. Our typical steady-state RANS simulations run for 5 h on 144 cores, and the time-dependent simulations run for 72 h on 192 cores; this is a reasonable and affordable computational cost even for a small-scale high-performance computing (HPC) facility. In a small-/medium-sized HPC infrastructure, many such simulations can run in parallel so that a lot of statistics can be collected simultaneously or large parametric studies can be conducted in a short time. Enhancing the quality of the air turbulence description through LES or DNS approaches makes the simulation too demanding to be used routinely for product design and optimization. However, such higher-fidelity simulations might be used to create and calibrate advanced turbulence models for the lighter RANS simulations, thus improving their quality. Alternatively high-fidelity simulation campaigns can be used to train fast reduced-order models designed to predict a specific physical property of interest [63].

A minor effect on particle deposition is apparently played by the type of inflow at the patient mouth; in fact, our calculations show how negligible the local deposition differences are when comparing a straight and a swirling inflow plume. Notice, however, that, in both cases, we are imposing the same flow splitting at the outlets by the mixed-driven boundary conditions described in the Materials and Methods section. While we know these flow splitting conditions are correct for the straight flow, as the latter inflow type was adopted in the SimInhale experiments in which they were measured, there is no a priori reason to expect that a swirled inflow would maintain them unaltered. If a different inflow type can give rise to different flow splitting, we must expect significant variations of local deposition, which we have summarized in the above paragraph and discussed in Section 3.3. Some authors [32,40,41,53,54,55] investigated deposition in the presence of two different inflow types with purely inlet-driven boundary conditions, i.e., the flow splitting can freely adjust along the bronchial tree, finding significant differences. Their results corroborate our hypothesis. However, a definitive confirmation requires dedicated in vitro experiments to at least measure the flow splitting in the presence of different inflow types. In any case, it is likely that none of the two flow driving conditions, adopted in simulations and in vitro experiments, is adequate to describe the correct flow splitting and deposition differences in physiologically plausible conditions.

A last point of attention resulting from our analysis is the fact that the particle splitting at each bifurcation does not necessarily follow the airflow one. Moreover, airflow splitting is far from being equally distributed between two daughter branches, contradicting the assumptions usually made in whole lung models or at least in the older but still widely adopted ones. CFD and CFD-DEM simulations could be used to provide better splitting rules, improving the level of accuracy of whole lung models.

**Figure 11:**
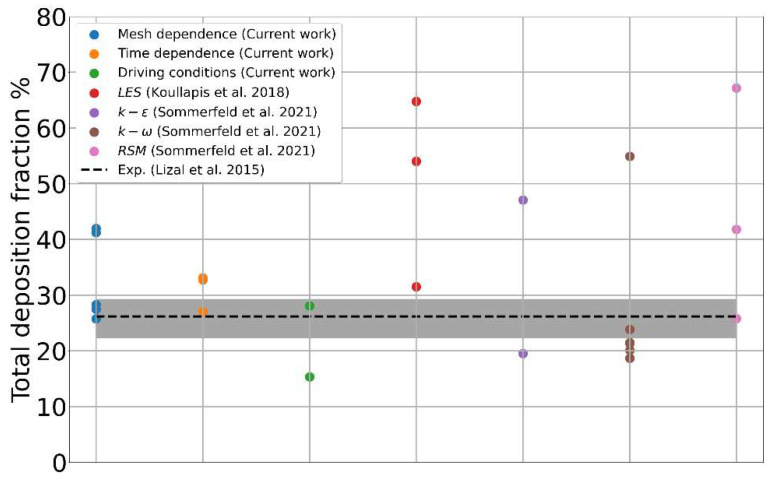
Summary of the computational results on the SimInhale model combining different numerical or physical ingredients. The first three columns summarize the aerosol deposition results presented in the previous sections. The blue cloud shows the total deposition fraction for the different meshes; the orange cloud displays the variability of the different time-dependent simulations, including those with particle–eddy interactions; and the green cloud compares the results obtained through various flow-driven boundary conditions. The other columns summarize the results obtained by other authors presenting different LES simulations [16] (red), RANS simulations with a different turbulence model [19] (purple and pink), and RANS simulations adopting our same turbulence model but a different meshing strategy [19] (brown). The black dashed line represents the experimental reference value, with its uncertainty bar in gray [17].

A direct comparison with both the experimental data and simulation results from other authors remains quite difficult, as the range of adopted flow rates and aerosol characteristics is very broad. Some of them study aerosol deposition in tidal breathing conditions, i.e., ±30 L/min, some others use a constant air inflow at the mouth, and still others apply realistic-like inspiratory profiles with a single peak reaching 60–80 L/min. In some simulations/experiments, the injected aerosol is monodispersed and previously diluted in the airflow entering the MT tract with continuity; in others, a cloud of particles/droplets with realistic polydisperse size distribution is released in a short time, as it usually occurs with DPI or MDI inhalers. Even restricting the comparison to simulations adopting the same geometry, similar airflow and aerosol conditions, the variability remains large due to different methodological and numerical issues highlighted by our analysis. The histogram of Figure 11 summarizes the simulation results for the total deposition published so far on the SimInhale reference geometry. The first three clouds of points summarize the variability of the results presented in the previous sections of this work; the remaining columns display the variability encountered by other authors. From the large scattering of the simulation results, it appears clearly how most of the uncertainty is not due to the choice of the turbulence model or the accuracy in its description but to the other numerical ingredients, i.e., mesh resolution, flow-driven boundary conditions, and time dependence. If follows that the calibration of an ad hoc turbulence model, based on currently missing experimental or DNS data characterizing the turbulent-to-laminar transition in the deeper airways, makes sense only after having sorted out these most impactful factors. Lastly, once the airflow turbulence will be correctly modeled, the focus will move to adequately capture the particle–eddy interaction and, more generally, the interaction of turbulence with the transported aerosol phase. Only when the dependence of the regional deposition on spatial discretization will be fully understood, and significantly reduced, it will make sense to quantify the deposition differences due to intersubject variability, different diseases, or different severities [64,65].

The definition of some standard rules and guidelines for the simulation of aerosol deposition would certainly reduce the observed large variability in the results and favor comparisons among different simulation works. To further investigate the problem of quantitative local deposition prediction, more local deposition data in controlled experimental conditions (e.g., using anatomically realistic 3D-printed casts) are desirable, perhaps involving particles/droplets of different sizes and natures and different inflow conditions.

## Data Availability

The raw data supporting the conclusions of this article will be made available by the authors on request.

## Supplementary Materials

The following supporting information can be downloaded at https://www.mdpi.com/article/10.3390/pharmaceutics16091119/s1: Figure S1: Convergence tests for regional particle deposition as a function of particle size, injection algorithm and injected particle number; Figure S2: Convergence tests for regional particle deposition grouping by lobes and sub-lobes.
