# Supplementary material for: A Critical Analysis of the CFD-DEM Simulation of Pharmaceutical Aerosols Deposition in Upper Intra-Thoracic Airways: Considerations on Aerosol Transport and Deposition
Source: Pharmaceutics. 2024 Aug 24;16(9):1119. doi: 10.3390/pharmaceutics16091119 (PMC11434992; doi:10.3390/pharmaceutics16091119)
Supplement: Supplementary file 1 [file pharmaceutics-16-01119-s001.zip › pharmaceutics-3133757-supplementary.pdf]

A critical analysis of the CFD-DEM simulation of pharmaceutical aerosols deposition in upper intra-thoracic airways: considerations on aerosol transport and deposition

Georgi H. Spasov [1,2], Riccardo Rossi [3], Andrea Vanossi [1,2], Ciro Cottini [4] and Andrea Benassi [1,4,*]

1 International School for Advanced Studies (SISSA), 34136 Trieste, Italy
2 Consiglio Nazionale delle Ricerche-Istituto Officina dei Materiali (CNR-IOM), 34149 Trieste, Italy
3 RED Fluid Dynamics, 09127 Cagliari, Italy
4 Chiesi Farmaceutici S.p.A., Largo Belloli, 11A, 43122 Parma, Italy
* Corresponding Author address: Chiesi Farmaceutici S.p.A. Largo Belloli 11A– 43122 Parma (Italy)
e-mail address: a.benassi@chiesi.com phone: +39 05211689162

Supplementary Material

1. Dependence of aerosol deposition on mesh resolution

The lack of convergence for the calculated aerosol deposition with increasing mesh resolution is discussed in section 3.1 of the main paper. Suppl. Figure S1 provides further evidence of how the deposition efficiency histograms are indeed well converged and robust upon

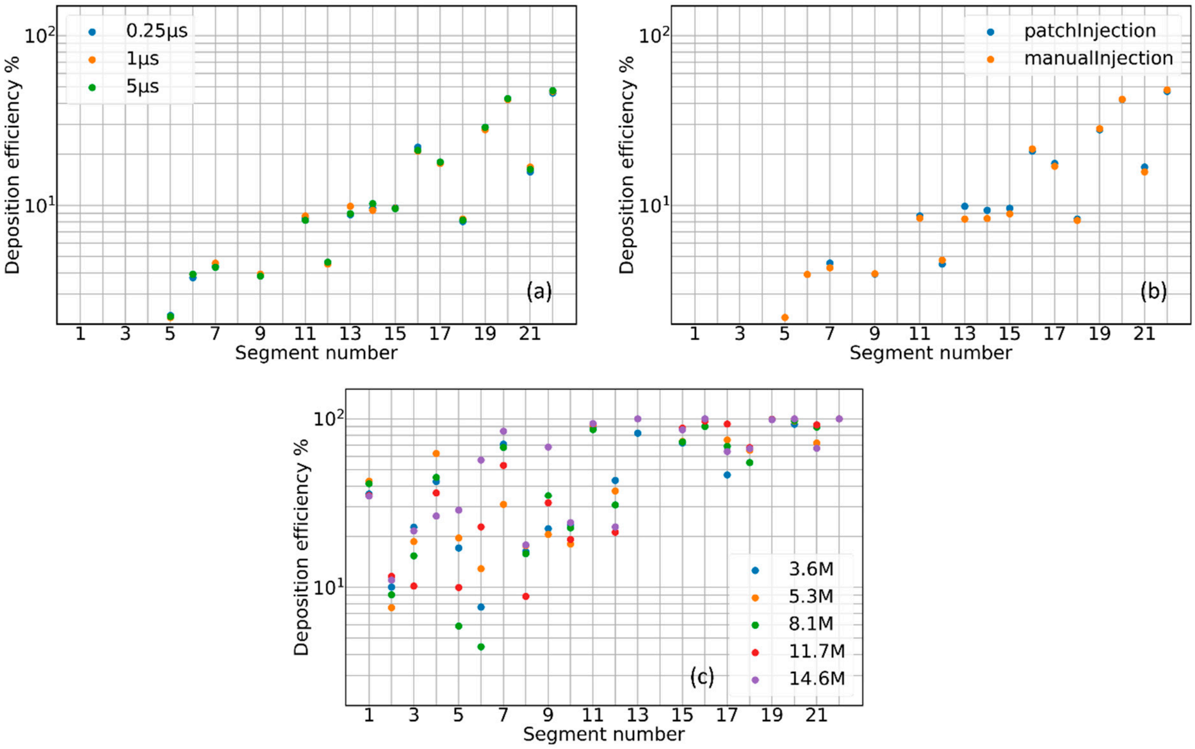

Figure S1: (a) percent deposition efficiency as a function of the segment index for the 8.1M elements mesh with different time integration steps Δt_p*. (b) percent deposition efficiency as a function of the segment index for the 8.1M for the two different particle injection mechanisms described in the Numerical Methods section. (c) percent deposition efficiency as a function of the segment index for* $10\ \mu m$ *particles with the 5 different meshes adopted in our study. Segment indexing is shown in Figure 1 (c) of the main paper.*

variation of other numerical details of the simulations. Panel (a) shows how the results are independent from the choice of the time integration step, indeed they are all small compared

to the smallest particle relaxation time estimated in section 2.3 of the main paper. Panel (b) documents how the deposition statistics is also robust against changes in the particle insertion algorithms (described in the Numerical Methods section). Finally, panel (c) reveals how the lack of convergence persists even increasing the size of the injected particles. Here the deposition efficiency histograms for particles with 10 μm diameter are plotted for all the mesh seizes, the lack of a converging trend with increasing mesh resolution is evident, similarly to what is presented in Figure 2 (c) of the main paper for 4.3 μm diameter particles. Investigation for larger particle diameters is meaningless as most of them tick in the mouth-throat tract being unable to reach the deeper segments.

2. Integrating aerosol deposition statistics

To regroup the deposition data by generation rather than by geometry segment, as in Figure 5 (b), one must associate a specific generation to each of the sticked particles. This is done through the following procedure:

1) A skeletonization of the bronchial tree geometry is performed starting from the stl surface file, using the CGAL libraries (https://www.cgal.org/). This procedure provides a non-centered skeleton of the geometry in the form of consecutive segments, such structure is shown in Figure 1 (b), its sections colorized according to airway generation number. Each segment is composed of starting and ending points, it is thus easy to define which segments pertain to the same generation and when a bifurcation occurs. If in the segments lists a point appears twice it is an internal point, if it appears three times it is a bifurcation point. Starting from the first point in the mouth, which appears only once, an iterative procedure can be applied to map the airway generation number on the skeleton.
2) Having the coordinates of the sticking point for each particle it is easy to calculate the distance ℓ between the sticking site and the skeleton. The latter is estimated as the smallest distance between the sticking site and all the skeleton points belonging to a given skeleton section. The sticked particle is then labelled by that specific airway section number and generation. When the sticking site is close to a bifurcation the same procedure is repeated for all the three skeleton sections composing the bifurcation and the sticked particle will be labelled by the section having the smallest ℓ value.

A further integration step is obtained by summing up the aerosol particles deposited in the sections pertaining to the same sub-lobe, or to the same lobe, leading to the deposition fraction histograms of Suppl. Figure S2.

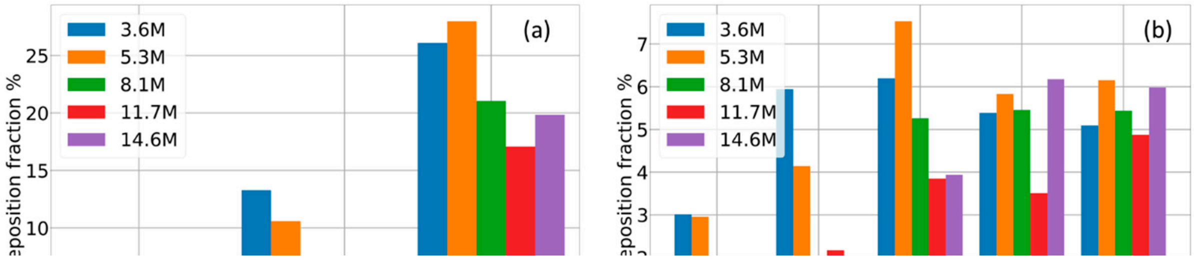

Figure S2: (a) percent deposition fraction for the different mesh sizes, grouped by mouth-throat trac (MT) and for left and right lobes. (b) percent deposition fraction for the different mesh sizes, grouped by sub-lobes (left-upper LU, left-lower LL, right-upper RU, right-middle RM and right-lower RL).

Many times in the main paper we refer to a percent uncertainty attributed to the aerosol deposition predictions as a function of the mesh size. This quantity can be calculated for the $j-th$ column of each deposition histogram as:

$$E_j = 100 \cdot \max_i \left[\frac{x_{ij} - \langle x \rangle_j}{\langle x \rangle_j}\right]$$

where $\langle x \rangle_j$ is the average over the different mesh sizes of the j-th column of deposition fraction DF_j or deposition efficiency DE_j as defined in the main paper; x_{ij} is the height of the $j-th$ column for the $i-th$ mesh histogram; the maximum value of the variance calculated for each $i-th$ mesh is finally taken. Notice that only the three bigger meshes, i.e. 8.1M, 11.7M and 14.6M elements, are considered in the uncertainty estimation.
